# The Adaptive Landscape of Genetic Interaction Network Has No Impact on Yeast Adaptive Evolution

**DOI:** 10.3389/fgene.2021.640501

**Published:** 2021-03-18

**Authors:** Joanna Klim, Urszula Zielenkiewicz, Anna Kurlandzka, Szymon Kaczanowski

**Affiliations:** ^1^Department of Microbial Biochemistry, Institute of Biochemistry and Biophysics, Polish Academy of Sciences, Warsaw, Poland; ^2^Department of Genetics, Institute of Biochemistry and Biophysics, Polish Academy of Sciences, Warsaw, Poland; ^3^Department of Bioinformatics, Institute of Biochemistry and Biophysics, Polish Academy of Sciences, Warsaw, Poland

**Keywords:** adaptive landscape, genetic interactions, compensatory evolution, *Saccharomyces cerevisiae*, yeast genetics

## Introduction

The extent of predictability of adaptive evolution and the impact of environmental conditions and genetic background on the evolutionary trajectory constitute fundamental questions of biology. It has been shown that non-lethal deleterious mutations are widespread in populations and are frequently accompanied by other mutations in the genome, suggesting compensatory evolution (Estes and Lynch, 2003; Doniger et al., 2008; Kenigsberg et al., 2010; MacArthur et al., 2012).

In recent years Pal's and Desai's groups employed *S. cerevisiae* deletion mutants as a model of compensatory evolution (Szamecz et al., 2014; Echenique et al., 2019). Those seminal studies indicated that the fitness losses due to gene deletions were rapidly compensated for by mutations elsewhere in the genome. In particular, frequent nonsense mutations were observed. It is expected that such mutations cause a loss of gene function and that the gene inactivation should have a positive impact on fitness. This expectation was tested experimentally. The Pal's group showed that a deletion of the *RPB9* gene was compensated by the inactivation of the *WHI2* gene and this observation was confirmed for diverse ecological conditions (Szamecz et al., 2014). On the other hand, a high-throughput study performed by Costanzo et al. (2016) allowed establishing a nearly complete genetic interaction network of *S. cerevisiae*. This network represents the fitness of a double mutant with two genes mutated or deleted in comparison with the fitnesses of the two single mutants which, in turn, are compared with the fitness of the wild-type ancestor. Usually, the impact of a pair of mutations is additive, and when there is no interaction—there is no epistasis. It has been demonstrated that the genetic interaction network has a modular structure and modules are established by genes involved in the same processes. What is more, the genetic interaction networks highlight cross-connection between biological processes, providing a global view on complex mechanisms of genetic inheritance of phenotypes.

We noticed that the genetic interaction networks containing fitness data of single and double mutants show whether in the background of a given deletion mutant a deletion of another gene is beneficial or deleterious, i.e., a fitness landscape. It is to be expected that the fitness landscape governs the process of evolution of deletion mutants (Weinreich et al., 2005). According to the genetic network data, the evolution of a given deletion mutant may involve inactivation of another gene(s) if such inactivation is beneficial for the mutant. In contrast, purifying selection removes loss-of-function mutations in genes whose inactivation is deleterious in the genetic network. Assuming that the genetic interaction network is stable it is expected that the landscape resulting from the network has an impact on the trajectory of compensatory evolution in similar conditions and similar genetic backgrounds. In this paper, we asked if the above-mentioned expectation is true and if indeed the fitness landscape of the genetic interaction network governs the process of adaptive evolution following gene deletion.

## Materials and Methods

The evolutionary landscape was obtained from Costanzo et al. (2016). Experimental evolutionary trajectories following systematic gene deletions were obtained from Szamecz et al. (2014) and Echenique et al. (2019). Those experiments were performed using yeast deletion mutants in BY4741 background grown in YPD medium and the starting genome sequence was determined following gene deletion to take into account possible off-target mutations caused by genetic manipulation. The data were analyzed using our own script based on Linux commands presented in Supplementary Dataset 1. Calculations of the dN/dS ratio for nonsense and missense mutations are presented in Supplementary Dataset 2.

## Results

The yeast genetic network described by Costanzo et al. was used as a model of the fitness landscape. It is the most comprehensive analysis of genetic interactions in which loss-of-function alleles for nearly all yeast genes were combined pairwise to generate almost 18 million double mutant strains. Using these data, we checked if the loss-of-function mutations found in experimental evolution, and believed to be compensatory, do indeed have a beneficial impact on the deletion mutant subjected to the experiment. We assumed that the loss of function of a second gene has a statistically significant impact on the fitness of the original deletion mutant if the absolute value of the difference between the fitness of the single and the double deletion mutant is higher than three times the standard deviation of the fitness of the double mutant. We found that for most gene pairs the effect was neutral (statistically insignificant), but in 2.6 million cases a second gene deletion increased the fitness of the original deletion mutant and in 3.3 million cases the second deletion decreased the fitness of the original mutant. We then inspected the results of compensatory evolution of gene deletion mutants observed by Szamecz et al. and Echenique et al. to establish how they related to the above fitness landscape. We posited that truncation and frameshift mutations caused inactivation of the gene basing on the observation of Szamecz et al. who proved that such mutations cause a loss of gene function. As shown in Table 1, truncation/frameshift of genes appearing during the evolution of deletion mutants usually had no impact on fitness. However, there are a few examples in which a gene inactivation was deleterious, e.g., deletion of the *STE50* gene for the *vps29*Δ strain (Supplementary Dataset 3). A list of affected genes and their possible impacts is presented in Supplementary Dataset 4.

**Table 1:** Effects of experimentally identified mutations on fitness according to fitness landscape.

		**Szamecz et al. (2014)**	**Echenique et al. (2019)**
Evolved deletion strains subjected to sequencing		14	22
Mutated genes (all types of mutations)	Total identified (non-silent mutations)	166	140
Putative loss-of-function mutations (truncation or frameshift)	Total identified	28 (12 represented in fitness landscape^†^)	35 (14 represented in fitness landscape^†^)
	Decreasing fitness according to gene interaction network	0	4
	Increasing fitness according to gene interaction network	1	0
	Neutral according to gene interaction network	11	10

† *Information about the fitness of the original mutant tested and the corresponding double mutant provided in Costanzo et al. (2016)*.

As shown in Table 1, inactivation of only one gene out of 26 represented in the fitness landscape was beneficial (ca. 4%). Supplementary analysis (Supplementary Dataset 2) revealed that the probability of beneficial inactivation of a random gene observed by Szamecz et al. and Echenique et al., according to the landscape, is 5 and 6%, respectively. According to binomial test, *P*-value that one gene is beneficial in the set of 26 genes is ~0.33. This means that observed frequency of beneficial gene truncations is random. Next, we estimated that the ratio of non-synonymous nonsense mutations to synonymous, dNONSENSE/dS is ~3.6 (Supplementary Dataset 2, part A). Thus, there are 3.6 times more than expected gene truncations under a neutral accumulation of mutations. Assuming that there are truncations that are neutral and beneficial, the frequency of neutral truncations is 1×dS, and beneficial is 2.6×dS. This indicates that ~70% of the nonsense mutations in the genome could be genuine driver mutations (2.6/3.6). This value is significantly greater than the number of beneficial putative loss of function mutations according to the landscape (*P* < 0.00000001, binomial test).

## Discussion

Results of experimental evolution indicate that the fitness landscape based on the genetic interaction network has no impact on the compensatory evolution of yeast deletion mutants. The frameshift mutations or truncation of genes observed during compensatory evolution of deletion mutants were usually neutral according to the fitness landscape; occasionally, they were deleterious and only very rarely—beneficial.

The experiments described in Szamecz et al. and Echenique et al. were performed in the same genetic background (BY4741), and the strain used in Costanzo et al., S288C, is the parental strain of BY4741. Also, the experimental conditions were similar in all three studies. Yeast were cultivated in YPD (yeast extract, peptone, glucose) rich medium. In the study on genetic interactions (Costanzo et al., 2016) fitness was gauged by colony size. Although compensatory evolution was performed in liquid culture, the compensatory effect of evolution was in the majority of cases confirmed by comparing colony sizes (Szamecz et al., 2014).

At first glance, the results presented above suggest that gene truncations are likely to be mostly neutral, as indicated by the fitness landscape. However, the experimental evolution study of Szamecz et al. provides different observations suggesting that gene truncations are usually compensatory by causing a loss of gene function. They are extremely frequent in comparison with synonymous (genuinely neutral) mutations and dN/dS approach indicates that the majority of them are beneficial. Additionally, that study showed that in parallel experiments on the evolution of the same deletion mutant, non-synonymous mutations often affect the same genes. Again, among such mutations, nonsense ones were extremely frequent (five out of a total 17). Furthermore, in two independent experiments, the *KIP1* gene was truncated during the evolution of *bim1*Δ strain (Szamecz et al., 2014) (Supplementary Dataset 3). Additionally, in the dataset of Echenique et al., the majority of nonsense mutations occur in sterile (*STE*) genes and four of them are in *STE11*. Interestingly, truncations of *STE11* took place during compensatory evolution following deletions of three different genes.

The apparent lack of an impact of the fitness landscape on the course of experimental compensatory evolution may also suggest that the mutations investigated to create the fitness landscape had different impacts than the mutations occurring during compensatory evolution. In the former case, gene deletions were analyzed while in the latter gene truncations were observed. However, at least in the case of compensation of *rpb9*Δ by deletion of *WHI2* the impact of both types of mutations has been shown to be quite similar. It is worth adding that Filteau et al. (2015) in a recent study also concluded that both genetic background and ecological conditions have an impact on the evolutionary trajectory.

To sum up, fitness measurements performed in yeast double knockout experiments are not predictive of compensatory evolution in experimental evolution lines, even if the experimental conditions (media and fitness measurements) and genetic background are similar.

The findings presented above suggest that the landscape of compensatory mutations is unstable and small perturbations such as mutations and/or small changes of ecological conditions cause changes of the landscape structure. We are unable to propose a waterproof explanation of this phenomenon, which suggests that compensatory evolution following gene inactivation is unpredictable. This conclusion has wide consequences as compensatory evolution likely is a basic mechanism of evolution of biological systems.

## Author Contributions

SK and UZ designed research. SK and JK analyzed data. JK, UZ, AK, and SK wrote the paper. All authors contributed to the article and approved the submitted version.

## Conflict of Interest

The authors declare that the research was conducted in the absence of any commercial or financial relationships that could be construed as a potential conflict of interest.
