# Supplementary material for: The Adaptive Landscape of Genetic Interaction Network Has No Impact on Yeast Adaptive Evolution
Source: Front Genet. 2021 Mar 18;12:640501. doi: 10.3389/fgene.2021.640501 (PMC8013701; doi:10.3389/fgene.2021.640501)
Supplement: Supplementary file 1 [file Data_Sheet_1.PDF]

Dataset S1. Detailed description of the analysis pipeline and a brief description of utilized datasets.

```
#This script uses as input files:
#
# 1. SGA_NxN.txt (genetic network)
# 2. csaba.tab (results obtained by group of Csaba Pal)
# 3. desnai.tab (results obtained by group of Michael Desai)

# and produces as output files:

# csaba.truncated_genes_ASMF Information about single mutant fitness
#obtained from Array SMF column (Set of Pal)
# csaba.truncated_genes_QSMF Information about single mutant fitness
#obtained from Query SMF column (Set of Pal)
# desnai.truncated_genes_ASMF Information about single mutant fitness
#obtained from Array SMF column (Set of Desai)
# desnai.truncated_genes_QSMF Information about single mutant fitness
#obtained from Query SMF column (Set of Desai)



# I PRE-EDITING

#1. Pre-editing. Extracting gene names from the network of Constanzo.
#Results are written in new_file  SGA_NxN1.txt
perl pp SGA_NxN.txt > SGA_NxN1.txt

# II ANALYSIS OF CSABA PAL SET

#  1.  Extracting information about interaction between deleted gene
#and gene with compensatory mutation. Data obtained from Csaba set in
#tabulated format is used.
#       Analysis is based on sort and merge algorithm, applied widely
#in relation databases.

#     a.   Creating an additional column in format
#gene_name_1_gene_name2 eg. YAL002WYBL001C in the network file.
#Systematic names are used. xResults are written in new file
#SGA_NxN2.txt
awk '{printf "%s%s %s\n",$1,$3,$0}'  SGA_NxN1.txt > SGA_NxN2.txt
#     b.   Sorting values of first column of the network file. Results
#are written in new file SGA_NxN2.txt.sort
sort -k1,1  SGA_NxN2.txt > SGA_NxN2.txt.sort
#     c.   Creating an additional column in format
#gene_name_1_gene_name2 in csaba.tab file eg. YAL002WYBL001C. Results
#are written in file csaba1.tab
awk '{printf "%s%s %s\n",$1,$8,$0}' csaba.tab >csaba1.tab
#d Sorting values of key (gene_name_gene_name)
sort -k1 csaba1.tab >csaba1.sort
#     d.   Joining Csaba.tab  and genetic interaction network. Note
#that files with sorted additional keys are used: SGA_NxN2.txt.sort
#and csaba1.sort
join -1 1 -2 1 csaba1.sort SGA_NxN2.txt.sort >
csaba1.sort_SGA_NxN2.txt.sort
#     e.   Extracting information about fitness impact of deletion of
#truncated gene during compensatory evolution according to Array SMF
awk '$0~/STOP|FRAME/&&$0!~/SYNONYMOUS/ ' csaba1.sort_SGA_NxN2.txt.sort >
csaba1.truncated_genes_ASMF
```

```
# 2. Extracting information about interactions between gene with
#compensatory mutation and deleted genes. Please note that
#interactions are not always ideally symmetrical
#    and sometime there are information about interaction gene a with
#gene b, but not of gene b with gene a
#    a. Creating the file of csaba input set with additional column in
#format gene_name_2_gene_name1. Please note that the order of columns
#is reversed. Results are written in file csaba1.tab.reverse.
awk '{printf "%s%s %s%s %s\n",$8,$1,$1,$8,$0}' csaba.tab
>csaba1.tab.reverse
#    b. Sorting results obtained in the previous step. Results are
#written to the file csaba1.tab.reverse.sort
sort -k1.1 csaba1.tab.reverse >csaba1.tab.reverse.sort
#    c.  Joining csaba.tab with inversed order of gene names and
#genetic interaction network. Note that files with sorted additional
#keys are used: SGA_NxN2.txt.sort and csaba1.tab.reverse.sort
join -1 1 -2 1 csaba1.tab.reverse.sort SGA_NxN2.txt.sort >
csaba1.sort_SGA_NxN2.txt.sort.reverse



# 3. Extracting unique information about interactions obtained in step
#number 2, which are not described by interactions in step number 1
#. a. Estracting a file containing gene pairs in interaction network
#obtained in step 1. Results are stored in file k1
awk '{print $1}' ../csaba1.sort_SGA_NxN2.txt.sort         |sort -u >k1
#. b. Estracting a file containing gene pairs in interaction network
#obtained in step 1. Results are stored in file k2
awk '{print $2}'    csaba1.sort_SGA_NxN2.txt.sort.reverse |sort -u >k2
#. finding unique gene pairs in k2, results are stored in k3
diff k2 k1 |awk '$1 ~/</ {print $2}' >k3
#  c. Finding subnetwork of interactions between mutated and deleted
#genes, which are described exclusively by interactions between genes
#with compensatory mutation and deleted genes.
grep -f k3 csaba1.sort_SGA_NxN2.txt.sort.reverse
>csaba1.sort_SGA_NxN2.txt.sort.reverse.unique
#  d. Extracting information about fitness impact of deletion of
#truncated gene during compensatory evolution according to Query SMF
#column
awk '$0~/STOP|FRAME/&&$0!~/SYNONYMOUS/ '
csaba1.sort_SGA_NxN2.txt.sort.reverse.unique > csaba.truncated_genes_QSMF

# III ANALYSIS OF DESAI SET
#  1.  Extracting information about interaction between deleted gene
#and gene with compensatory mutation. Data obtained from Desai set in
#tabulated format is used.
#       Analysis is based on sort and merge algorithm, applied widely
#in relation databases.

#     a.   Creating an additional column in format
#gene_name_1_gene_name2 eg. vps8ecm15 in the network file. Standard
#names are used instead of systematic (like in case of Csaba Pal set)
#          set  Results are written in new file 3SGA_NxN2.txt
awk '{printf "%s%s %s\n",$2,$4,$0}'  SGA_NxN1.txt > SGA_NxN3.txt
#     b.   Sorting values of the first column of the network file.
#Results are written in new file SGA_NxN3.txt.sort
sort -k1,1 SGA_NxN3.txt > SGA_NxN3.txt.sort
```

```
#     c.   Creating an additional column in format
#gene_name_1_gene_name2 in desai.tab file eg. ADE2ADE4. Results are
#written in file desai1.tab
awk ' {printf "%s%s %s\n",$1,$4,$0 } '  desnai.tab >desai1.tab
#d Sorting values of key (gene_name_gene_name)) and transforming this
#key into lower letters
sort -k1,1 desai1.tab|awk ' {print tolower ($0)} '  > desai1_sort
#     d.   Joining desai.tab  and genetic interaction network. Note
#that files with sorted additional keys are used: SGA_NxN3.txt.sort
#and desai1.sort
join -1 1 -2 1 desai1_sort SGA_NxN3.txt.sort  >
desai_sort1SGA_NxN3.txt.sort
#     e.   Extracting information about fitness impact of deletion of
#truncated gene during compensatory evolution according to Array SMF
awk '$6~/\*/||$7~/nt\)/' desai_sort1SGA_NxN3.txt.sort >
desai.truncated_genes_ASMF

# 2. Extracting information about interactions between gene with
#compensatory mutation and deleted genes. Please note that
#interactions are not always ideally symmetrical
#    and sometime there are information about interaction gene a with
#gene b, but not of gene b with gene a
#    a. Creating the file of csaba input set with additional column in
#format gene_name_2_gene_name1. Please note that the order of columns
#is reversed. Results are written into file desai1.tab.reverse.
awk ' {printf "%s%s %s%s %s\n",$4,$1,$1,$4,$0 } '   desai.tab
>desai1.tab.reverse
#    b. Sorting results obtained in the previous step. Letters are
#transformed into lower cases. Results are written to the file
#desai1.tab.reverse.sort
sort -k1.1 desai1.tab.reverse | awk ' {print tolower ($0)} '
>desai1.tab.reverse.sort
#    c.  Joining desai.tab  (with inversed order of gene names) and
#genetic interaction network. Note that files with sorted additional
#keys are used: SGA_NxN3.txt.sort and desai1.tab.reverse.sort
join -1 1 -2 1 desai1.tab.reverse.sort SGA_NxN3.txt.sort >
desnai1.sort_SGA_NxN3.txt.sort.reverse

# 3. Extracting unique information about interactions obtained in step
#number 2, which are not described by interactions in step number 1
#. a. Extracting a file containing gene pairs in interaction network
#obtained in step 1. Results are stored in file k1
awk '{print $1}' desai_sort1SGA_NxN3.txt.sort    |sort -u >k1
#. b. Extracting a file containing gene pairs in interaction network
#obtained in step 1. Results are stored in file k2
awk '{print $2}' desai1.sort_SGA_NxN3.txt.sort.reverse|sort -u >k2
#. finding unique gene pairs in k2, results are stored in k3
diff k2 k1 |awk '$1 ~/</ {print $2}' >k3
#  c. Finding subnetwork of interaction between mutated and deleted
#genes, which are described exclusively by interactions between genes
#with compensatory mutations
grep -f k3 desai1.sort_SGA_NxN3.txt.sort.reverse
>desai1.sort_SGA_NxN3.txt.sort.reverse.unique
#  d. Extracting information about fitness impact of deletion of
#truncated gene during compensatory evolution according to Query SMF
#column
awk '$7~/\*/||$8~/nt\)/ ' desai1.sort_SGA_NxN3.txt.sort.reverse.unique >
desai.truncated_genes_QSMF
```

```
#This script is used for calculation of probability that deletion of
gene will have beneficial, deleterious #or neutral impact on fitness
of deletion mutants used in experiments by Csaba Pal and Michael
Desai #group according to genetic network.


#This script uses as input files:
# 1. SGA_NxN.txt (genetic network)
# 2. csaba.tab (results obtained by group of Csaba Pal)
# 3. desai.tab (results obtained by group of Michael Desai)

#I. Analysis of Csaba Pal data set
#Analysis is based on merge and sort algorithm
#1. Preparation of the sorted list of non-redundant deleted genes in
#experiment of Csaba Pal group.
#Genes names are also transformed to lower cases.
awk ' {print $1}' csaba.tab |sort -u >csaba_lista
#2  Sorting genes names in the network
sort -k1,1 SGA_NxN1.txt > SGA_NxN1.txt.sort
#3 "Joining" non redundant list of genes obtained in point number 1
#with  "Query strain id". In result,
#obtained file contains exclusively "Query strain ID" of genes used
#in experiments
join -1 1 -1 1 SGA_NxN1.txt.sort csaba_lista >
csaba_lista.SGA_NxN1.txt.sort
#4. Additional column containing impact of gene deletion expressed
as #ZSCORE is added. Results are
#saved as csaba_lista.SGA_NxN1.txt.sort_zscore
awk ' $NF>0&&$(NF-3)~/[0-9]/&&$(NF-1)~/[0-9]/ {print
$0,($(NF-1)-$(NF-3))/$NF} ' csaba_lista.SGA_NxN1.txt.sort >
csaba_lista.SGA_NxN1.txt.sort_zscore
#5. Calculation fraction of beneficial and deleterious gene
deletions #for deletion mutants from experiment of Csaba Pal
awk ' {a=a+1;if($NF>3) {b=b+1}; if($NF<-3) {c=c+1}} END {print
"number of gene deletions",a,"number of beneficial gene
deletions",b,"number of deleterious gene deletions",c, "fraction of
deleterious gene deletions",c/a,"fraction of beneficial gene
deletions", b/a}' csaba_lista.SGA_NxN1.txt.sort_zscore
#6 Removing working files
rm csaba_lista.SGA_NxN1.txt.sort_zscore
csaba_lista.SGA_NxN1.txt.sort SGA_NxN1.txt.sort

#II. Analysis of Desai data
#Analysis is based on merge and sort algorithm
```

```
#1. Preparation of the sorted list of non-redundant deleted genes in
#experiment of Desai group.
# Genes names are also transformed to lower cases.
awk ' {print tolower($1)}' desai.tab |sort -u >desai_lista
#2  Sorting genes names in the network
sort -k2,2 SGA_NxN1.txt > SGA_NxN1.txt.sort
#3 "Joining" non redundant list of genes obtained in point number 1
#with  Query allele names. In result, obtained file contains
#exclusively "Query allele names" of genes used in experiments by
#Desai group (deleted in founder populations). Results ares saved as
#desnai_lista.SGA_NxN1.txt.sort
join -1 2 -2 1 SGA_NxN1.txt.sort desai_lista >
desai_lista.SGA_NxN1.txt.sort
#4. Additional column containing impact of gene deletion expressed
as #ZSCORE is added. Results are saved as
#desnai_lista.SGA_NxN1.txt.sort_zscore
awk ' $NF>0&&$(NF-3)~/[0-9]/&&$(NF-1)~/[0-9]/ {print
$0,($(NF-1)-$(NF-3))/$NF} ' desai_lista.SGA_NxN1.txt.sort >
desai_lista.SGA_NxN1.txt.sort_zscore
#5. Calculation fraction of beneficial and deleterious gene
deletions #for deletion mutants from experiment of desai
awk ' {a=a+1;if($NF>3) {b=b+1}; if($NF<-3) {c=c+1}} END {print
"number of gene deletions",a,"number of beneficial gene
deletions",b,"number of deleterious gene deletions",c, "fraction of
deleterious gene deletions",c/a,"fraction of beneficial gene
deletions", b/a}' desai_lista.SGA_NxN1.txt.sort_zscore
#6 Removing working files
rm desai_lista.SGA_NxN1.txt.sort_zscore
desai_lista.SGA_NxN1.txt.sort SGA_NxN1.txt.sort
```
