# Supplementary material for: The Adaptive Landscape of Genetic Interaction Network Has No Impact on Yeast Adaptive Evolution
Source: Front Genet. 2021 Mar 18;12:640501. doi: 10.3389/fgene.2021.640501 (PMC8013701; doi:10.3389/fgene.2021.640501)
Supplement: Supplementary file 2 [file Data_Sheet_2.PDF]

**Dataset S2**

**Part A** – Details of dN/dS calculations for synonymous and missense mutations ratio.

| | Szamecz *et al.* (2014) | Echenique *et al.* (2019) |
|---|---|---|
| Synonymous mutations | 25 | no data |
| Synonymous sites (possible synonymous mutations in mutated genes) | 141224 | no data |
| Synonymous substitution rate (dS) (substitutions/synonymous site) | 0.00018 | no data |
| Non-synonymous mutations | 138 | 105 |
| Non-synonymous sites (possible non-synonymous mutations) | 485092 | 372646 |
| Non-synonymous substitution rate (dN) (non-synonymous substitutions/non-synonymous sites) | 0.00029 | 0.00028 |
| dN/dS | 1.6 | no data |
| Nonsense mutations | 21 | 13 |
| Nonsense sites (possible nonsense mutations) | 32871 | 23652 |
| Nonsense substitution rate (dNONSENSE) (nonsense substitutions/nonsense sites) | 0.00063 | 0.00055 |
| dNONSENSE/dS | 3.6 | no data |
| dNONSENSE/dN | 2.2 | 1.96 |

**Part B** - The script used for calculation of a number of synonymous, missense and nonsense sites.

```
It uses as an input the fasta files of mutated genes in Desai and Csaba
experiments.
#skrypt_nonsense_ds.python is our python program.
python skrypt_nonsense_ds.python desai.fasta
python skrypt_nonsense_ds.python csaba.fasta

#The code for the skrypt_nonsense_ds.python is presented below:


from Bio.Seq import Seq
from Bio import SeqIO
import os
import io
```

```
import sys

print(sys.argv[1])

#with open(sys.argv[1]) as f:
#      print(f)

def gene_nonsense_ds(input_dna):
    synonymous_number=0
    nonsense_number=0
    non_synonymous_number=0
    dna_code=str("ACTG")
    input_bialko=Seq(input_dna).translate()
    for i in range(0,len(input_dna)):
    #mutated_str=input_dna
        #print(dna_code)
        for i1 in dna_code:
            mutated_str=list(input_dna)
            if input_dna[i]!=i1:
                mutated_str[i]=i1
                mutated_dna="".join(mutated_str)
                mutated_bialko=Seq(mutated_dna).translate()
            #print(mutated_dna,mutated_bialko)
                if mutated_bialko==input_bialko:
                #print("synonymous")
                #print(mutated_dna,mutated_bialko)
                   synonymous_number=synonymous_number+1
            #print(wynik1)
                if mutated_bialko.count("*")>0:
                #print(mutated_bialko.count("*"))
                #print(mutated_dna,mutated_bialko)
                    nonsense_number=nonsense_number+1
                if  mutated_bialko!=input_bialko:
                    if mutated_bialko.count("*")==0:
                         #print("mutacja")
                         #print(mutated_dna,mutated_bialko)
                         non_synonymous_number=non_synonymous_number+1
    return(synonymous_number,nonsense_number,non_synonymous_number)

records=list(SeqIO.parse(sys.argv[1],"fasta"))

for record in records:
        test_protein=str(record.translate().seq)
        #print(test_protein)
        #print(test_protein.count("*"))
        if str(test_protein).count("*")>0:
                   print("error")

output=[0,0,0]
for record in records:
    #print(record)
    result=gene_nonsense_ds(str(record.seq))
    output[0]=output[0]+result[0]
    output[1]=output[1]+result[1]
    output[2]=output[2]+result[2]

print("number of possible synonymous mutations",output[0])
print("number of possible nonsense mutations",output[1])
```

```
print("number of possible non synonymous mutations",output[2])
```
