# Supplementary material for: The Adaptive Landscape of Genetic Interaction Network Has No Impact on Yeast Adaptive Evolution
Source: Front Genet. 2021 Mar 18;12:640501. doi: 10.3389/fgene.2021.640501 (PMC8013701; doi:10.3389/fgene.2021.640501)
Supplement: Supplementary file 4 [file Data_Sheet_4.pdf]

Dataset S4. Genes bearing putative loss-of-function mutations (truncation or frameshift) identified in Szamecz et al., (2014) and Echenique et al., (2019) experiments.

Truncation/nonsense	Frameshift
Szamecz *et al*., (2014)	
ARP3, ART5, CAT8, DNM1, GEA1, HXT13, IRA1, KIP1, PAT1, PBP1, PUF4, RFC1, RIM15, RSC30, SRB8, UBX6, WHI2, YOR1, YCR025C, YMR010W	*CSM3, MNC1, PEX6, SUN4, TMN3, TEL1, YKR040C*
Echenique *et al*., (2019)	
ADE6, IRA1, PIL1, STE4, STE5, STE11, STE50, PAN2, PDE2	*ADE4, BBC1, CIN8, CLA4, EAP1, FAB1, IRA1, ISW1, LAA1, MEI5, NUP192, PDE2, STE4, STE11, STE7, SWH1, TBS1, UTR2, YNG2*

In both data sets, we identified over-representation of genes involved directly or indirectly in genome stability and ploidy maintenance: *CIN8, CSM3, EAP1, ISW1, KIP1, MEI5* (this was unexpected because this gene encodes a meiosis-specific protein), *PAN2, PAT1, PDE2, RIM15, RSC30, RFC1* (although the significant 2/3 truncation of the latter should be lethal), *SRB8, TEL1, UBX6,* and *YNG2*. This can be easily explained because genome instability usually leads to changes in ploidy which in yeast are evolutionarily beneficial. Another over-represented group of genes comprised those linked to metabolism or nutrient-sensing: *HXK2, HXT13, IRA1 ART5, PBP1, YOR1, CAT8, COX6,* and *COX4*. We assume that the inactivation of these genes could be linked to reducing unnecessary processes in the given environmental conditions.

It has to be noticed that some nonsense mutations identified by Echenique *et al*. (2019) were compensated for by mutations in other genes. For instance, a mutation in *ADE2* was compensated by *ade6*. The accumulation of the red pigment in *ade2* cells is likely to cause an impaired growth. However, *ade6* is epistatic to *ade2* and blocks adenine biosynthesis before the accumulation of the red pigment, resulting in a white cell phenotype (Roman (1956, 1957)). Some truncations studied by Echenique *et al*., (2019) e.g., *PEP8, VPS29, ERG3, SOK2,* and again *ADE2* were compensated for by mutations in the *STE4, STE5, STE7, STE11,* or *STE50* genes linked to stress response, MAP kinases signaling, and pheromone response and mating pathways. It cannot be excluded that the compensatory mutations in these genes led to a mating-type switch and in consequence allowed diploidization.
